# Advances in drug delivery system for platinum agents based combination therapy

**DOI:** 10.7497/j.issn.2095-3941.2015.0063

**Published:** 2015-12

**Authors:** Xiang Kang, Hai-Hua Xiao, Hai-Qin Song, Xia-Bin Jing, Le-San Yan, Ruo-Gu Qi

**Affiliations:** ^1^Department of Obstetrics and Gynecology, Union Hospital, Tongji Medical College, Huazhong University of Science and Technology, Wuhan 430022, China; ^2^State Key Laboratory of Polymer Physics and Chemistry, Changchun Institute of Applied Chemistry, Chinese Academy of Sciences, Changchun 130022, China; ^3^University of Chinese Academy of Sciences, Beijing 100049, China; ^4^Department of General Surgery, Ruijin Hospital, Shanghai Jiao Tong University School of Medicine, Shanghai 200025, China; ^5^Shanghai Minimally Invasive Surgery Center, Shanghai 200025, China

**Keywords:** Cancer, drug delivery, combination therapy, platinum

## Abstract

Platinum-based anticancer agents are widely used as first-line drugs in cancer chemotherapy for various solid tumors. However, great side effects and occurrence of resistance remain as the major drawbacks for almost all the platinum drugs developed. To conquer these problems, new strategies should be adopted for platinum drug based chemotherapy. Modern nanotechnology has been widely employed in the delivery of various therapeutics and diagnostic. It provides the possibility of targeted delivery of a certain anticancer drug to the tumor site, which could minimize toxicity and optimize the drug efficacy. Here, in this review, we focused on the recent progress in polymer based drug delivery systems for platinum-based combination therapy.

## Introduction

Platinum-based anticancer agents are widely used as first-line drugs in cancer chemotherapy for various solid tumors, such as testicular cancer, bladder cancer, ovarian cancer, non-small cell lung cancer (NSCLC), small cell lung cancer (SCLC), melanoma, and lymphomas^1^^,^^2^. Cisplatin (cis-diamminedichlo-platinum, CDDP) is the first approved platinum drug that has been used for more than three decades in standard chemotherapy regimens^3^. Due to the low concentration of chloride, cisplatin is hydrolyzed inside the cell and converted to the highly reactive species [Pt(NH_3_)_2_Cl(OH_2_)]^+^, which forms 1,2-GpG intrastrand adducts with DNA. The adducts inhibit transcription and replication of DNA, ultimately leading to cellular apoptosis^4^^-^^6^. However, the use of cisplatin is restricted because of its severe side effects, including nephrotoxicity, neurotoxicity, ototoxicity, and myelo-suppression, as well as the intrinsic and acquired resistance developed by various cancers^7^^-^^12^. For this reason, improved Pt-based anticancer drugs that display mitigated side effects and are able to overcome one or more resistance mechanisms have been developed^13^^,^^14^. Carboplatin, oxaliplatin, nedaplatin, lobaplatin, and heptaplatin that have similar structure to cisplatin are now clinically used (**Table 1**)^25^. In addition, new types of compounds, such as prodrugs [platinum (IV)] that can be directly reduced to Pt(II) in the cancer cells^26^ and multinuclear and intercalating complexes, show promising advantages both *in vitro* and *in vivo*^27^^,^^28^. Nevertheless, side effects and occurrence of resistance remain the major drawbacks for nearly all the developed Pt drugs. To solve these problems, new strategies should be adopted for Pt drug-based chemotherapy.

**Table 1:** Pt complexes in clinical use

Formulation	Status	Year approved	Structure	Indications	Reference
Cisplatin	Worldwide clinical use	1979	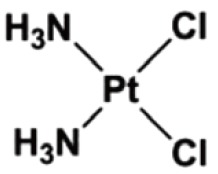	Testicular cancer, ovarian cancer, bladder cancer, head and neck cancer, NSCLC, SCLC, gastric cancer, anal cancer	^6^
Carboplatin	Worldwide clinical use	1989	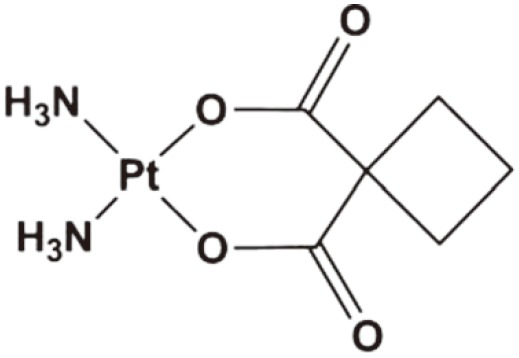	Ovarian cancer, NSCLC, SCLC, melanoma, head and neck cancer, thymic cancer, breast cancer	^15^^,^^16^
Oxaliplatin	Worldwide clinical use	2002	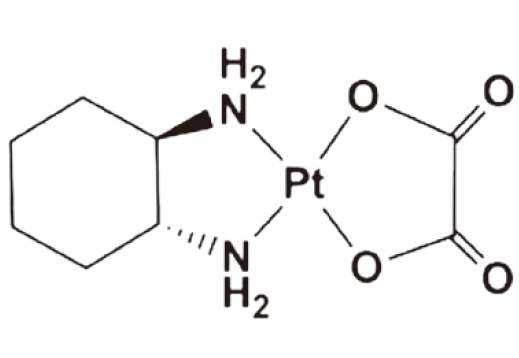	Colorectal cancer	^17^^-^^19^
Nedaplatin	Clinical use in Japan	1996	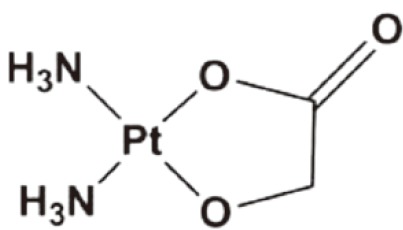	NSCLC, SCLC, esophageal cancer, head and neck tumors, bladder cancer	^20^^,^^21^
Lobaplatin	Clinical use in China	2004	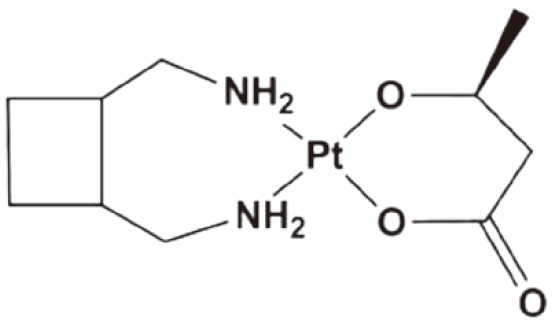	Breast cancer, SCLC, chronic myeloid leukemia	^22^
Heptaplatin	Clinical use in South Korea	2005	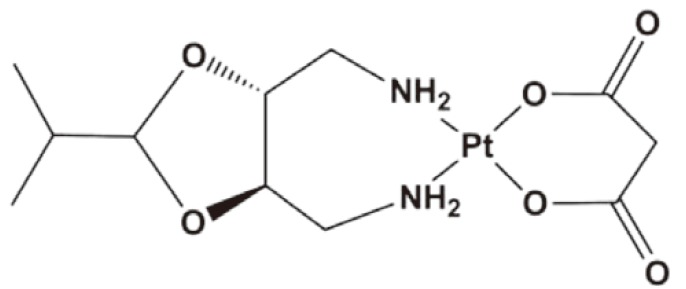	Gastric cancer	^23^^,^^24^

The first strategy is combination therapy described as the simultaneous administration of two or more pharmacologically active agents with different mechanisms. Combination therapy has long been adopted as a primary cancer treatment regimen^29^. Compared with single-drug therapy, combined therapy is capable of reducing drug resistance by targeting different signal pathways^30^. Moreover, this approach may offer higher therapeutic efficacy via synergistic effects than the single-drug therapy^31^. However, different drugs vary in solubility, pharmacokinetics, biodistribution, and membrane transport mechanisms; hence, designing dosage and schedule of regimen are difficult. Furthermore, these different drugs sometimes bring even more serious side effects. In particular, almost all of the Pt drugs used in clinics nowadays share similar mechanism of action as DNA cross linking agents. **Table 2** lists various Pt-based combination regimens used in clinics for multiple targets in the cancer cells.

**Table 2:** Most commonly used Pt-based combination regimens suggested by NCCN

Drug/regimen	Indications	Dosage and schedule (could be variable)
Cisplatin, fluorouracil (PF)	Anal, bladder, cervical, esophageal, gastric, and head and neck cancer	Cisplatin 75-100 mg/m^2^ IV on days 1 and 29; Fluorouracil 750-1,000 mg/m^2^ IV continuous infusion over 24 h daily on days 1-4 and 29-32; 35-day cycle
Cisplatin, etoposide (EP/PE)	NSCLC, SCLC, ovarian, prostate, testicular, thymic, and neuroendocrine cancer	Cisplatin 80 mg/m^2^ day 1 and etoposide 100 mg/m^2^ days 1, 2, 3
Carboplatin, etoposide (EC)	SCLC, ovarian, prostate, testicular, and neuroendocrine cancer; soft tissue sarcoma	Carboplatin AUC 5-6 day 1 and etoposide 100 mg/m^2^ days 1, 2, 3
Carboplatin, paclitaxel (TC/TP)	Breast, cervical, ovarian, endometrial, esophageal, gastric, thymic, and thyroid cancer; melanoma; NSCLC	Paclitaxel 175 mg/m^2^ IV over 3 h followed by carboplatin AUC 56 IV over 1 h day 1 repeat every 3 weeks × 6 cycles
Oxaliplatin, leucovorin, fluorouracil (FLOFOX)	Colorectal cancer	Oxaliplatin 85 mg/m^2^ IV over 2 h, day 1; leucovorin 400 mg/m^2^ IV over 2 h, day 1; 5-FU 400 mg/m^2^ IV bolus on day 1, and then 1,200 mg/m^2^/day × 2 days (total 2,400 mg/m^2^ over 46-48 h) IV continuous infusion; repeat every 2 weeks
Oxaliplatin, capecitabine (CAPOX/XELOX)	Colorectal, esophagus, and gastric cancer	Oxaliplatin 130 mg/m^2^ IV over 2 h, day 1; capecitabine 850-1,000 mg/m^2^ twice daily PO for 14 days; repeat every 3 weeks

The second strategy is the use of nanotechnology. Modern nanotechnology has been widely employed in the delivery of various therapeutics and diagnostics^32^^-^^36^, thereby providing the possibility of targeted delivery of a certain anticancer drug to the tumor site to minimize toxicity and optimize drug efficacy^37^^,^^38^. Nanoparticle delivery system can improve drug solubility, reduce systemic toxicity, increase blood circulation time, enhance cell uptake, and provide controllable release profiles^38^^-^^41^. Moreover, the system can even deliver the drugs to selective cancer cells either by taking advantage of enhanced permeability and retention effect (EPR effect) or by specific bimolecular recognition between nanoparticles and cells^42^^,^^43^. Nanoparticle delivery system for single drugs has been successful with pioneer examples, such as Abraxane and Doxil. The drugs are currently used in clinics. Nonetheless, single-drug therapy is rarely used in clinical practice because combination therapy is more compelling, as previously described^29^. However, the real challenge for combination therapy is reproducing the *in vitro* synergy effect *in vivo* because different drugs have different metabolism profiles. The drug ratio resulting in synergy *in vitro* may not translate to synergy *in vivo* because the combined drugs are transported into the tumor cells differently, making it a barrier for ideal combination therapy. Nonetheless, nanoparticle has the ability to simultaneously encapsulate/conjugate one, two, or multiple drugs at a desirable drug ratio. Furthermore, combined drugs can be delivered to the ultimate target at the initial ratio with minimal leakage because the nanoparticles protect the drugs^44^. Hence, scientists around the world develop nanoparticle drug delivery systems for combination chemotherapy.

Specifically, numerous drug delivery carriers, such as polymer, solid lipid and inorganic nanoparticles, have been developed over the years for the delivery of single Pt drugs. Thus, much improvement has been made in this field^44^. For example, polymer-Pt(II) conjugates, such as AP5280 (cisplatin) and AP5346 (oxaliplatin), entered phase II clinical study^45^^,^^46^. NC-6004, the polyglutamic acid-Pt(II) drug developed by Kataoka is now used in clinics, as well as in a phase II study in Japan and in a phase III study in the USA^47^. A more advanced example is lipoplatin, which has successfully completed a phase III trial for non-small cell lung cancer in 2010^48^^,^^49^. Developments in single Pt drug delivery system have been successful. Meanwhile, these drug carriers are also developed for Pt-based combination therapy. This review focuses on the recent progress in polymer-based drug delivery systems for Pt-based combination therapy.

## Polymeric drug delivery system for Pt agent based combination therapy

### General combination strategy

The general combination strategy for free drugs is relatively simple. The delivery of a specific drug, however, can be either conjugated or encapsulated. Moreover, the drug conjugate in aqueous solution can either form nanoparticles or not. **Table 3** summarizes the general combination strategies and their advantages and drawbacks with consideration of the complexity of the drug delivery system.

**Table 3:** General combination therapy strategy for two anticancer drugs

Categories	Combination strategy	Advantages/drawbacks
Conjugate A+ free B	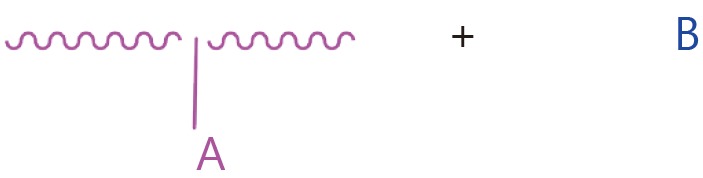	Starting drug ratio tunable; difference in metabolism
Conjugate A+ conjugate B	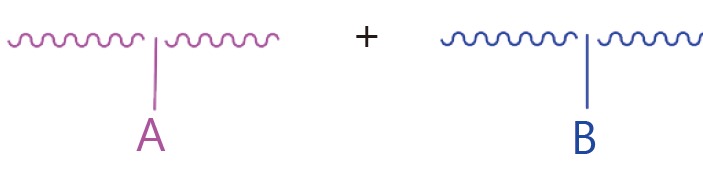	Starting drug ratio tunable; difference in metabolism
A and B co-conjugate	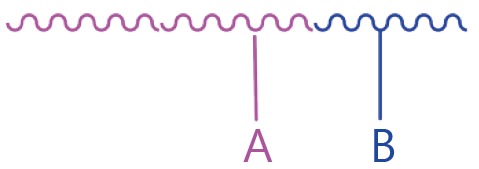	Starting drug ratio tunable; similar metabolism
A-NPs+ free B	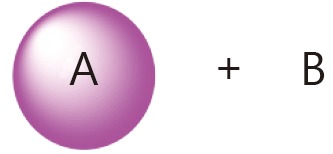	Starting drug ratio tunable; difference in metabolism; minimum change drug ratio at circulation
A-NPs+ B-NPs	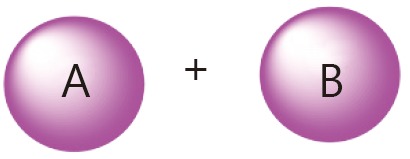	Drug ratio tunable; difference in metabolism;
A and B co-loaded NPs	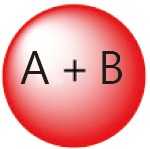	Starting drug ratio tunable; difference in metabolism; minimum change drug ratio at circulation

### Pt(II)-based combination

Drug sensitizers are commonly employed, including the combination of paclitaxel, docetaxel, doxorubicin (DOX), 5-FU and gemcitabine, and Pt agents such as oxaliplatin, cisplatin, or carboplatin, to improve the efficacy of chemotherapy, combination therapies of Pt(II) agents, and other agents, e.g., anticancer drugs.

**Figure 1:**
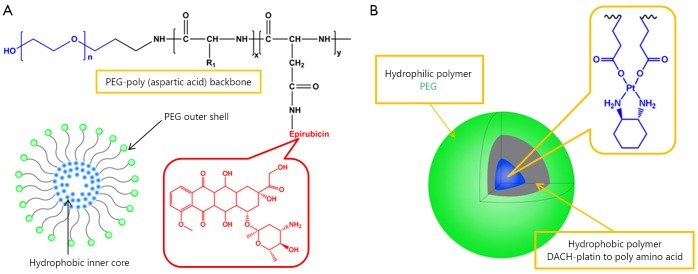
Representative drug combinations of two drug loaded nanoparticles. (A) Combined NC-6003 (epirubicin). (B) NC-4016 (oxaliplatin) micelles.

Epirubicin has been widely used for several cancer types^2^. NC-6300 is an epirubicin-containing micelle in which epirubicin is covalently bound to polyaspartate block copolymer through an acid-labile hydrazone bond with a diameter of 40 to 80 nm, as shown in **Figure 1A** ^50^. As described above, NC-4016 is a polyethylene glycol-poly (glutamic acid) block copolymer, PEG-P (Glu) micelle formed via complex of DACHPt, and carboxyl groups in the polymer side chains (**Figure 1B**). NC-6300 and NC-4016 micelles are combined and compared to the free drug combination of oxaliplatin and epirubicin (E/O) in 44As3Luc cells and *in vivo* xenografts^51^. The combined two nanoparticles display highly synergistic effect equivalent to E/O. *In vivo*, the nanoparticle combination generates greater antitumor activity in the subcutaneous tumor model, which results in longer life span of the animals, compared with the free drug combination. Furthermore, the nanoparticle combination has less cardiotoxicity and neurotoxicity, which can be beneficial for future clinical trials.

**Figure 2:**
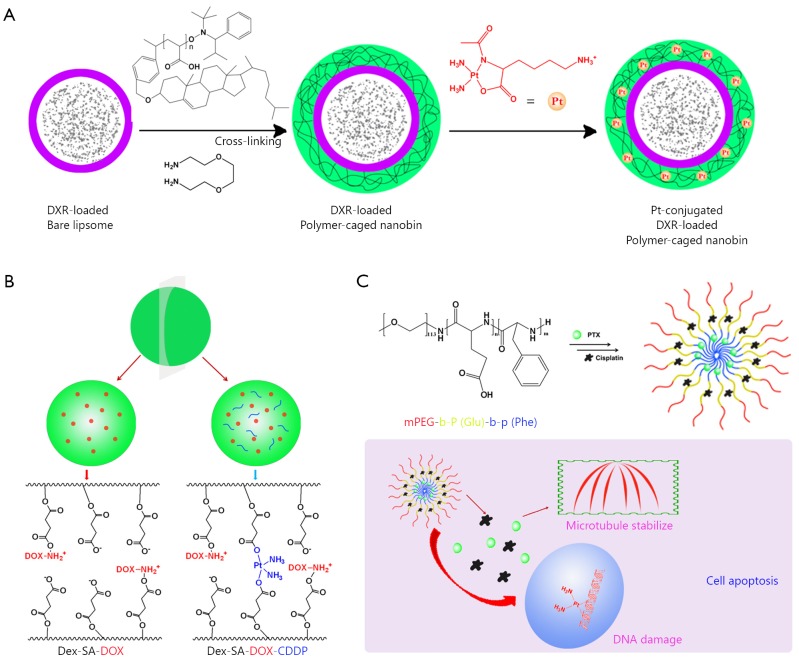
Combination of two strong cytotoxic agents in one nanoparticle platform. (A) Doxorubicin and cisplatin prodrug were combined in a polymer-caged nanobin. (B) Cisplatin and doxorubicin were combined in dextrin-derivative polymer micelles. (C) Paclitaxel and cisplatin were combined in poly-peptide nanomicelles.

Apart from the combination of two separate micelles, incorporating multi-components in one regimen has become very common in clinical practice. Therefore, the combination of two or more cytotoxic anticancer agents in one nanoscale delivery platform for cancer chemotherapy becomes fascinating. Lee *et al.*^52^ proposed a way of co-packaging doxorubicin and cisplatin in a single polymer caged nanobin (PCN). This PCN had a doxorubicin-encapsulated liposomal core and a pH-responsive cisplatin prodrug loaded polymer shell. Moreover, the drug ratio, surface charge, and zeta potential in the PCN can be adjusted. PCN showed stronger synergy than the free drug combination (**Figure 2A**) and extensively enhanced the overall cytotoxicity. This example demonstrated the future potential of nano-platform for multi-drug combinational delivery.

Li *et al.*^53^ reported that pH-responsive cisplatin prodrug crosslinked polysaccharide-based nanoparticles. These nanoparticles were developed from succinic acid decorated dextran (Dex-SA) for loading and triggered intracellular release of DOX (**Figure 2B**). Further crosslinking the nanoparticles by Pt(II) agents resulted in crosslinked nanoparticles. Different from conventional crosslinking agent, cisplatin was used as crosslinking agents in the said study. The *in vitro* release experiment demonstrated that DOX could be released from the crosslinked nanoparticles in a controlled/pH-dependent manner. The crosslinked nanoparticles had a relatively longer blood circulation half-life than free doxorubicin and un-crosslinked nanoparticles. Consequently, *in vivo*, the DOX-loaded CL-nanoparticles exhibited enhanced therapeutic efficacy in tumor-bearing mice compared with the non-CL-nanoparticles and free DOX, which were further confirmed by the histological and immunohistochemical analyses. These cisplatin prodrug crosslinked polysaccharide nanoparticles proved to be a promising nanomedicine drug delivery system for tumor-targeted delivery of DOX.

Thereafter, Dex-SA-DOX-CDDP nanoparticles were tested in three colorectal and breast mouse tumor models, namely, subcutaneous colorectal carcinoma xenograft, dimethylhydrazine induced autochthonous colorectal and metastatic mammary carcinoma^54^. These animal models could well mimic the pathological and immunological responses triggered by tumors in patients. The Dex-SA-DOX-CDDP nanoparticles inhibited the growth of CT26 xenograft tumors, possibly because of efficient tumor accumulation and penetration. More importantly, the life span was extended in an autochthonous colorectal carcinoma model. With the addition of iRGD, the growth and metastasis of 4T_1_ tumors were retarded. Therefore, Dex-SA-DOX-CDDP could be an effective platform for the delivery of DOX.

Paclitaxel and cisplatin were extensively combined in clinics and called TP regimen, as listed in **Table 2**. As shown in **Figure 2C**, Song *et al.*^55^ reported that a novel methoxy poly(ethylene glycol)-b-poly(L-glutamic acid)-b-poly (L-phenylalanine) [mPEG-b-P(Glu)-b-P(Phe)] triblock copolymer was prepared and explored as a micelle carrier for the co-delivery of paclitaxel (PTX) and cisplatin. PTX and CDDP were co-loaded into the micelles with the hydrophobic P(Phe) and P(Glu)-Pt block as the inner core and the PEG as the hydrophilic shell. *In vitro* drug release experiments showed that via cross-linking by CDDP, the burst release of PTX could be avoided. Moreover, this nanoparticle with both PTX and CDDP showed high synergy in the inhibition of A549 lung cancer cells over a 72 h period of drug incubation. *In vivo*, the PTX and CDDP co-loaded micelles demonstrated an obvious tumor suppression rate of 83.1%, which was significantly higher than that of free drug combination and single drug-loaded micelles. Moreover, this nanoparticle showed fewer side effects. Thus, the polypeptide-based combination of PTX and CDDP may provide useful guidance for effective and safe cancer chemotherapy.

**Figure 3:**
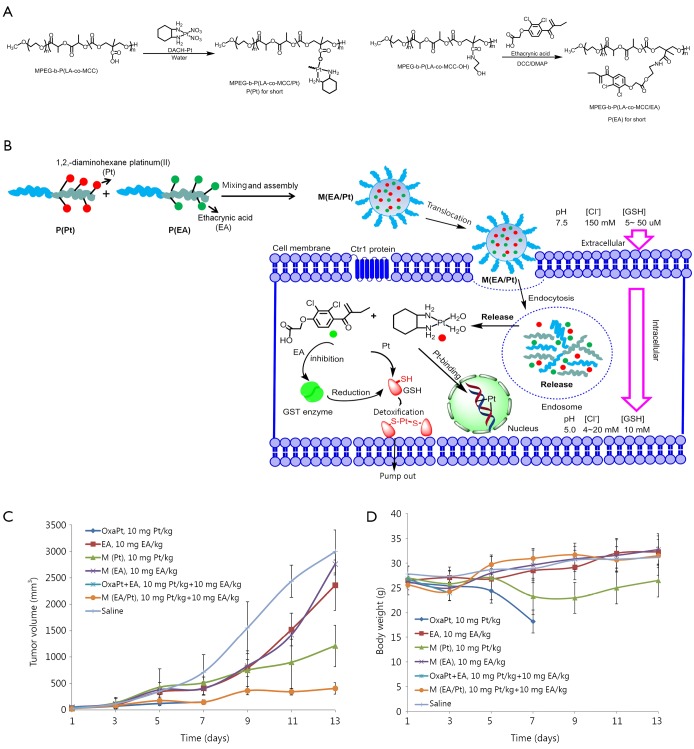
Combination of a cytotoxic platinum agent with a less cytotoxic drug sensitizer ethacrynic acid to minimize the glutathione mediated Pt detoxification. (A) Chemical structure of P(Pt) and P(EA). (B) Schematic illustration of self-assembling P(Pt) and P(EA) into M(EA/Pt) and its intracellular fate. (C) *In-vivo* evaluation of tumor inhibition effect of M(EA/Pt). (D) *In-vivo* evaluation of body weight change after treatment of M(EA/Pt).

The combination of two cytotoxic agents was introduced in the previous sections. With the possibility that two cytotoxic agents may cause more severe side effects, combining less cytotoxic agents (such as drug sensitizer, which has minimum toxicity and can sensitize the anticancer drug) may be more promising. The major drawback of Pt drugs is drug resistance. Notably, one of the major reasons for Pt drug resistance is GSH-mediated detoxification. Ethacrynic acid (EA), which is a GST inhibitor, is non-toxic and can reduce the conjugation of GSH with Pt agents to sensitize cancer cells to chemotherapy^56^. As shown in **Figure 3**, Yang *et al.*^57^ prepared a polymer conjugate of EA and then constructed biodegradable polymeric nanoparticles to co-deliver both EA and DACHPt (a precursor of oxaliplatin) to overcome GSH-mediated detoxification. Yang *et al.*^57^ first showed that the hybrid nanoparticles released EA and Pt faster in lower pH values. The nanoparticles with both EA and Pt showed synergistic effect, resulting in a 4.6-fold increase in anticancer efficacy. *In vivo* study also supported the ideas of these researchers. The EA used in their study was nearly non-toxic. By utilizing its sensitizing effect, synergy can be also generated. This approach is different from combining two cytotoxic agents together, which might cause greater side effects.

### Pt(IV) based combination

**Figure 4:**
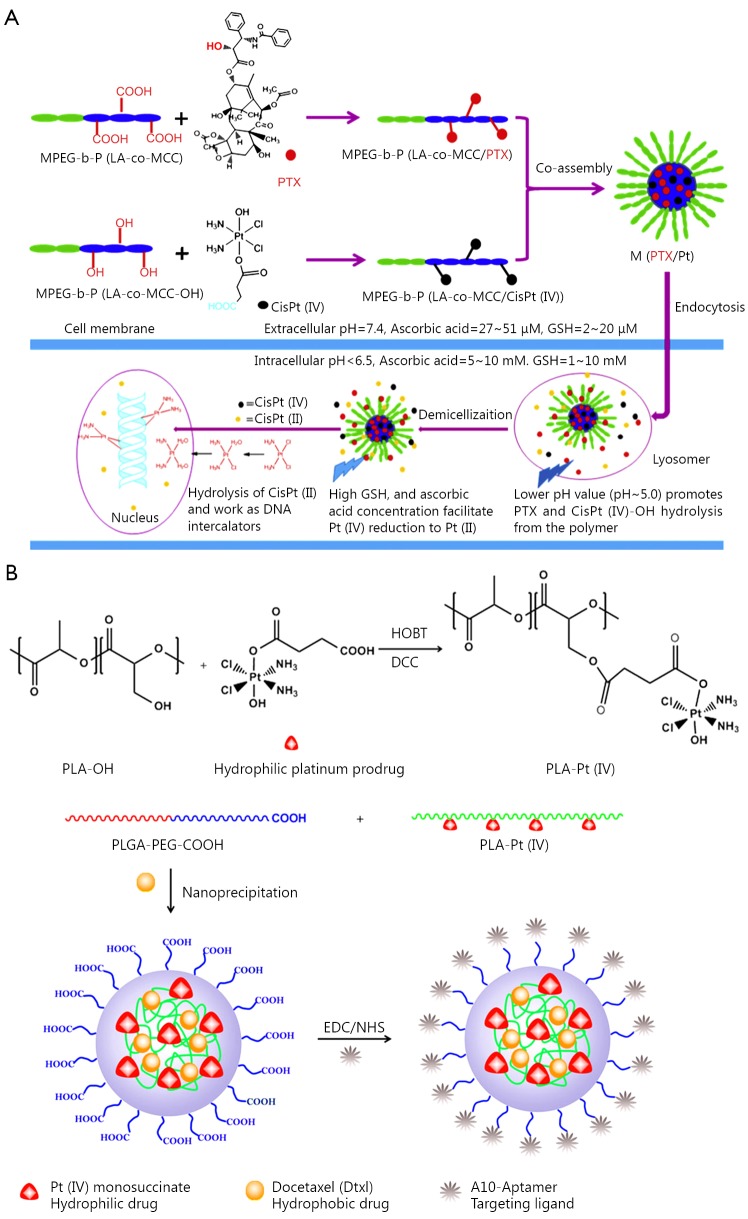
Combination of two cytotoxic agents in one nanoparticle platform. (A) Polymer-cisplatin (IV) conjugates and polymer-paclitaxel conjugates were mixed to prepare micelles with both cisplatin and paclitaxel for combinational drug delivery. (B) Polymer-cisplatin (IV) conjugates were used to encapsulate docetaxel for combinational drug delivery.

Pt(IV) drugs, prodrugs of Pt(II) agents, are more stable and can result in less side effects. Pt(IV) drugs can be modified to possess sufficient lipophilicity for drug encapsulation or to gain functional groups for drug conjugation. Though numerous reports on Pt(II) based drug combinations in a polymer platform are available, Pt(IV) drug-based combination therapy in nanoparticles was relatively underdeveloped. A method for delivering cisplatin(IV) prodrug and paclitaxel by using composite micelles was designed by Xiao *et al.*^58^ (**Figure 4A**). A cisplatin(IV) conjugate and a paclitaxel conjugate with the same biodegradable and amphiphilic block copolymer were synthesized and then co-assembled. The polymer-cisplatin(IV) conjugate had a Pt loading of 14% (w/w). The loading capacity of paclitaxel in the high polymer was 30% (w/w). By changing the ratio of the two conjugates, different proportions of Pt/PTX composite micelles were obtained. When the micelles entered the tumor cells, effective anticancer drug cisplatin(II) was released upon cellular reduction, and PTX was released by acid hydrolysis. Moreover, the composite micelles achieved synergy *in vitro* and *in vivo*. Therefore, the combination therapy could reduce systematic toxicity and enhance antitumor efficacy.

A self-assembled polymeric nanoparticle (NP) platform developed by Kolishetti *et al.*^59^ was used to reach the targeted tumor cells and precisely control the co-delivery of drugs with varying physicochemical properties (**Figure 4B**). Cisplatin and docetaxel (Dtxl) were delivered to prostate cancer cells with synergistic cytotoxicity. By preparing a polylactide (PLA) derivative with pendant hydroxyl groups and conjugating it to a Pt(IV) prodrug, a new functionalized polymer c,t,c-[Pt(NH_3_)_2_(O_2_CCH_2_CH_2_COOH)(OH)Cl_2_] [PLA-Pt(IV)] was obtained. This polymer was mixed with carboxyl-terminated poly(D, L-lactic-co-glycolic acid)-block-poly(ethylene glycol) copolymer in the presence or absence of Dtxl in microfluidic channels. Through this reaction, a new polymer with 95% encapsulation efficiency (EE), 5% loading of the hydrophilic Pt(IV) drug, 80% EE, 1% loading of hydrophobic Dtxl, and a diameter of 100 nm was successfully synthesized. The surface of these nanoparticles was modified with the aptamer A10 group, which could be specifically targeted to prostate-specific membrane antigen (PSMA) on prostate cancer cells. These nanoparticles could undergo controlled release of both drugs over a period of 48-72 h. The nanoparticles with targeting moieties were internalized into the PSMA-expressing LNCaP cells, and the formation of cisplatin 1,2-d(GpG) intrastrand cross-links was found. The *in vitro* toxicity experiments showed that the dual drug combined with target group was better than that of single drug nanoparticles or non-targeted NPs. This work reveals the possibility of combining two drugs in a single polymeric nanoparticle drug delivery system to treat tumors.

In the previous section, the combination of two cytotoxic agents was achieved either by mixing two separate drug conjugates or encapsulating a second drug with the polymer-drug conjugate of the first drug. This strategy could easily formulate nanoparticles with two drugs at a desirable ratio. However, this approach cannot ensure that the exact ratio of the drugs combined will remain unchanged during circulation because premature leaking of one drug might occur. Pt(IV) prodrugs have six coordinated positions, which can be utilized to attach other molecules, including anticancer drugs, drug sensitizers, imaging agents, and targeting ligands. Multifunctional Pt(IV) prodrugs with other anticancer drugs or drug sensitizers at a precise ratio can be designed for combinational therapy. These multifunctional Pt(IV) agents can be intracellularly reduced to release Pt(II) agents and the attached molecules.

**Figure 5:**
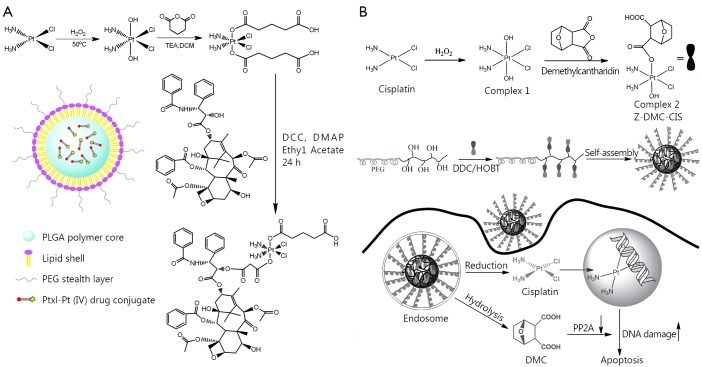
Ratio metric combination of two drugs in a multifunctional Pt(IV) prodrug. (A) Paclitaxel and cisplatin were combined in a Pt(IV) prodrug and encapsulated for drug delivery. (B) Norcantharidin and cisplatin were combined in a Pt(IV) prodrug and conjugated for drug delivery.

As shown in **Figure 5A**, Aryal *et al.*^60^ designed a multifunctional Pt(IV) agent with both cisplatin and paclitaxel in the same molecule for combination therapy. In this Pt(IV) molecule, paclitaxel and cisplatin were loaded at a precise ratio of 1:1. Moreover, the hydrophobicity of the molecule easily rendered encapsulation into lipid-polymer hybrid nanoparticles with controllable drug loading and desirable drug release profiles. The cytotoxicity of the multifunctional Pt(IV) drugs loaded nanoparticles on ovarian cancer cells was further evaluated in comparison with un-encapsulated free drug conjugates. The results demonstrated that encapsulation of the drug conjugate could greatly enhance cytotoxicity, possibly because of the facilitation of the intracellular entry of the amphiphilic drug conjugates across the lipid bilayer structured cellular membranes.

Serine/threonine protein phosphatase 2A (PP2A) is recognized as a promising therapeutic target for the treatment of cancer^61^. Traditional Chinese medicines, such as cantharidin or its derivative demethylcantharidin (DMC), are potent PP2A inhibitors^62^. Combination of PP2A inhibitors with other chemotherapy drugs, such as Pt agents, generated synergy. As shown in **Figure 5B**, Zhou *et al.*^63^ designed multifunctional Pt(IV) agents containing cisplatin and DMC (complex 1 in **Figure 5B**). Instead of being encapsulated with free carboxylic acid group into nanoparticles, this drug conjugate was conjugated to biodegradable polymer to prepare a polymer-(tandem drugs) conjugate drug delivery system (DLS size: 200-240 nm). The tandem drug delivery system has the following advantages: (I) precise ratio control of drugs delivered, which is hardly achieved by other methods; (II) simultaneous release of two drugs; and (III) high chance of reproducing the *in vitro* synergy *in vivo*. The designed polymer-(tandem drugs) conjugate was believed to act in a dual mode, i.e., reduction and release of Pt(II) agents could target the intracellular DNA, resulting in DNA double strand crosslinks and releasing DMC-induced DNA damages to bypass cell cycle checkpoints and lethal mitosis by inhibition of PP2A.

Similar multifunctional Pt(IV) drugs designed for drug delivery by our group are Z-DCA-Pt, S-DCA-Pt, and camplatin, which also showed their benefit of combination^64^^-^^66^.

## Conclusion and future perspective

Pt agents comprise 50% of all anticancer drugs used in clinics and in 80% of clinical anticancer regimens as a single agent or combined with other anticancer drugs. The delivery system for Pt(II) drugs was relatively better developed than that for Pt(IV) drugs because several Pt(II) drug delivery systems entered different clinical phases. More work should be done to enhance the characteristics of Pt(IV) agents to maximize benefits and identify the best drug delivery system. Both Pt(II) and Pt(IV) drug-based combination delivery systems were relatively popular in research, but not in clinical trials. However, in present clinical practice, the combination of Pt agents with one or more anticancer drugs is prevalent. Different profiles of pharmacokinetics, metabolism, and side effects would compromise the overall therapeutic outcomes of combination therapy. To resolve these problems, the transfer of this clinical combination in a drug delivery system may bring benefits despite the extremely challenging translation. The clinical benefits of combination therapy using nanoparticle platforms should be unambiguously demonstrated. Moreover, the superiority should be proven from the drug delivery rather than from the synergy generated by the parent drugs. Therefore, the advantages of delivering two or more drugs in one platform should be better than combining single drugs. The Pt drugs and their combinations are ubiquitous in clinical practice. Hence, the prospects of translation of the combination therapy into drug delivery system would be favorable.
